# Taking precautions in advance: a lower level of activities of daily living may be associated with a higher likelihood of memory-related diseases

**DOI:** 10.3389/fpubh.2023.1293134

**Published:** 2023-12-15

**Authors:** Jiawei He, Weijie Wang, Shiwei Wang, Minhua Guo, Zhenyan Song, Shaowu Cheng

**Affiliations:** ^1^School of Integrated Chinese and Western Medicine, Hunan University of Chinese Medicine, Changsha, China; ^2^Key Laboratory of Hunan Province for Integrated Traditional Chinese and Western Medicine on Prevention and Treatment of Cardio-Cerebral Diseases, College of Integrated Traditional Chinese and Western Medicine, Hunan University of Chinese Medicine, Changsha, China; ^3^School of Informatics, Hunan University of Chinese Medicine, Changsha, China

**Keywords:** activities of daily living, memory-related diseases, machine learning algorithm, the China health and retirement longitudinal survey, Barthel Index (BI)

## Abstract

**Introduction:**

Memory-related diseases (MDs) pose a significant healthcare challenge globally, and early detection is essential for effective intervention. This study investigates the potential of Activities of Daily Living (ADL) as a clinical diagnostic indicator for MDs. Utilizing data from the 2018 national baseline survey of the China Health and Retirement Longitudinal Study (CHARLS), encompassing 10,062 Chinese individuals aged 45 or older, we assessed ADL using the Barthel Index (BI) and correlated it with the presence of MDs. Statistical analysis, supplemented by machine learning algorithms (Support Vector Machine, Decision Tree, and Logistic Regression), was employed to elucidate the relationship between ADL and MDs.

**Background:**

MDs represent a significant public health concern, necessitating early detection and intervention to mitigate their impact on individuals and society. Identifying reliable clinical diagnostic signs for MDs is imperative. ADL have garnered attention as a potential marker. This study aims to rigorously analyze clinical data and validate machine learning algorithms to ascertain if ADL can serve as an indicator of MDs.

**Methods:**

Data from the 2018 national baseline survey of the China Health and Retirement Longitudinal Study (CHARLS) were employed, encompassing responses from 10,062 Chinese individuals aged 45 or older. ADL was assessed using the BI, while the presence of MDs was determined through health report questions. Statistical analysis was executed using SPSS 25.0, and machine learning algorithms, including Support Vector Machine (SVM), Decision Tree Learning (DT), and Logistic Regression (LR), were implemented using Python 3.10.2.

**Results:**

Population characteristics analysis revealed that the average BI score for individuals with MDs was 70.88, significantly lower than the average score of 87.77 in the control group. Pearson’s correlation analysis demonstrated a robust negative association (*r* = −0.188, *p* < 0.001) between ADL and MDs. After adjusting for covariates such as gender, age, smoking status, drinking status, hypertension, diabetes, and dyslipidemia, the negative relationship between ADL and MDs remained statistically significant (*B* = −0.002, *β* = −0.142, *t* = −14.393, 95% CI = −0.002, −0.001, *p* = 0.000). The application of machine learning models further confirmed the predictive accuracy of ADL for MDs, with area under the curve (AUC) values as follows: SVM-AUC = 0.69, DT-AUC = 0.715, LR-AUC = 0.7. Comparative analysis of machine learning outcomes with and without the BI underscored the BI’s role in enhancing predictive abilities, with the DT model demonstrating superior performance.

**Conclusion:**

This study establishes a robust negative correlation between ADL and MDs through comprehensive statistical analysis and machine learning algorithms. The results validate ADL as a promising diagnostic indicator for MDs, with enhanced predictive accuracy when coupled with the Barthel Index. Lower levels of ADL are associated with an increased likelihood of developing memory-related diseases, underscoring the clinical relevance of ADL assessment in early disease detection.

## 1 Introduction

In recent years, there has been a burgeoning interest in investigating the intricate relationship between Activities of Daily Living (ADL) and memory-related diseases (MDs), largely fueled by the burgeoning older-adults population (1). This subject matter has attracted substantial attention within the domains of medical and public health research (2–4). MDs, encompassing Alzheimer’s Disease (5), Parkinson’s Disease Dementia (6), Frontotemporal Dementia (7), Vascular Dementia (8), Huntington’s Disease (9), and Amyotrophic Lateral Sclerosis (10), impose formidable challenges on individuals, families, and healthcare systems due to their profound impact on cognitive faculties and overall well-being. Projections suggest that the number of dementia cases in China will escalate to a staggering 100 million by 2050 (11, 12). This looming surge in the dementia population portends significant burdens on individuals, families, and healthcare infrastructures. Therefore, it is imperative to comprehensively comprehend the factors underpinning memory-related ailments to ameliorate symptoms and forestall further deterioration.

MDs are characterized by progressive cognitive deficits that erode an individual’s memory retention capacity, cognitive processing abilities, and logical reasoning faculties (5). In tandem, ADL encompass a spectrum of facets related to an individual’s self-care, functional proficiency, and task-execution prowess (13), rendering them pivotal components of overall well-being and personal autonomy. A plethora of studies have unveiled a substantive correlation between ADL and disorders typified by memory impairment. For instance, individuals diagnosed with Alzheimer’s Disease may contend with not only deteriorating memory function but also challenges in maintaining personal hygiene, reduced engagement in social activities, and a loss of autonomy in daily living (14). Those who have experienced a stroke leading to hemiparesis may grapple with mobility issues, diminished self-care abilities, progressive cognitive decline, memory lapses, and potentially even incipient dementia symptoms (15). The core objective of clinical rehabilitation interventions hinges on addressing ADL as a foundational element in restoring patients’ self-sufficiency (16). This therapeutic paradigm seeks to mitigate the long-term consequences of ailments and enhance the overall prognosis of individuals undergoing rehabilitation. In tandem with the evolution of healthcare paradigms, the assessment of the health status of middle-aged and older-adult populations has transcended traditional metrics centered on disease incidence and mortality rates (17). Accumulating evidence posits ADL as a pivotal variable for evaluating and scrutinizing the physical well-being of middle-aged and older-adult cohorts, given its potential significance in precipitating dementia onset.

Given the gradual and insidious progression of MDs, there exists a pressing need to proactively seek prospective clinical indicators and bolster preventive measures. This study employs rigorous statistical analysis to prognosticate the association between ADL and MDs, leveraging data gleaned from the 2018 national baseline survey of the China Health and Retirement Longitudinal Study (CHARLS). Furthermore, it corroborates conventional statistical findings through the deployment of machine learning methodologies such as Support Vector Machine (SVM), Decision Tree (DT), and Logistic Regression (LR). The principal aim of this endeavor is to elucidate the nexus between ADL and memory-related disorders, with a view to enhancing early detection capabilities and tailoring interventions that improve the health and quality of life of individuals associated with MDs.

## 2 Methods

### 2.1 Study population

Our study draws upon data sourced from the CHARLS baseline dataset, meticulously administered by the National School for Development at Peking University.^1^ CHARLS, in its pursuit of a comprehensive understanding of the dynamics within the middle-aged and older-adult demographic, has embarked upon the creation of an impeccably curated, high-resolution developmental database. This repository constitutes an expansive repository of multifaceted data, comprising intricate details regarding individuals, their health statuses, familial backgrounds, and socio-economic dimensions, all meticulously compiled for each participant within the cohort.

The study cohort itself is composed of individuals aged 45 years and above, painstakingly selected through a stratified random sampling strategy that spans 150 counties or districts and encompasses 450 villages or urban clusters distributed across 28 provinces (18). This meticulous selection process ensures a faithful representation of the demography of the middle-aged and older-adult population across China, thereby capturing a nuanced cross-section of this demographic stratum.

Our investigation leverages the dataset derived from CHARLS for the year 2018, encompassing a total of 20,813 samples. Following a rigorous screening process, the final number of samples included in our study amounted to 10,062.

### 2.2 Assessment of memory-related disease

Memory-related diseases were assessed from the record of “Diagnosed with Memory-Related Disease by a Doctor” in the CHARLS data. We categorized the memory-related disease population into two groups: Yes (already diagnosed with a memory-related disease) or No (no memory-related disease).

### 2.3 Assessment of ADL

The evaluation of ADL is meticulously conducted through the utilization of the Barthel Index (BI), a widely recognized instrument renowned for its efficacy in assessing ADL (Table 1). The Barthel Index methodically categorizes a spectrum of routine activities into ten discrete elements, encompassing essential tasks such as Feeding, Bathing, Grooming, Dressing, Bowel Control, Bladder Control, Toilet Use, Transfers, Mobility, and Stairs. Each of these elements is assigned a score, which reflects the individual’s proficiency in performing the respective task. The cumulative scores from these items are aggregated to generate individual Barthel Index scores, with a potential score range of 0 to 100. Notably, higher scores on the Barthel Index signify elevated levels of autonomy and proficiency in the execution of activities of daily living (19–21).

**Table 1:** Activities of daily living scale.

Daily activities	Independent	Partially independent	Moderately dependent	Totally dependent
Feeding	10	5	0	0
Bathing	5	0	0	0
Grooming	5	0	0	0
Dressing	10	5	0	0
Bowel control	10	5	0	0
Bladder control	10	5	0	0
Toilet use	10	5	0	0
Transfers	15	10	5	0
Mobility	15	10	5	0
Stairs	10	5	0	0

### 2.4 Assessment of covariates

In the current study, we collected data on several covariates using the CHARLS questionnaire. These covariates encompassed age, gender, place of residence (Central City/Town, Urban–Rural Integration Zone, Rural, or Special Zone), educational attainment (Primary or below, Secondary school [Middle school + High school], College or above), smoking status (yes, quit, or no), drinking status (no, drink less than once a month, or drink more than once a month), as well as the presence of hypertension, diabetes, and dyslipidemia. The age groups were classified as middle-aged individuals, defined as those aged 45 years and older but less than 60 years, and older-adult individuals, defined as those aged 60 years and more. According to the 2010 Chinese guidelines for hypertension, hypertension was characterised as having a mean systolic blood pressure of 140 mm Hg or higher and/or a mean diastolic blood pressure of 90 mm Hg or higher, and/or self-reported use of antihypertensive medication within a two-week period (22). The definition of diabetes encompasses those with fasting blood glucose levels equal to or greater than 7.0 mmol/L, or those who are currently undergoing treatment with hypoglycemic medication. According to the 2016 Chinese guidelines for dyslipidemia in adults, dyslipidemia is diagnosed when certain lipid levels are seen. These levels include a total cholesterol level of 240 mg/dL or higher, a high-density lipoprotein cholesterol level of less than 40 mg/dL, a low-density lipoprotein cholesterol level of greater than 160 mg/dL, or triglyceride levels of 200 mg/dL or higher (23).

### 2.5 Statistical analysis

In the presentation of continuous variables, the mean and standard deviation (SD) were employed as summary statistics, while categorical variables were expressed as numbers and percentages. To examine demographic variations, the independent sample t-test was utilized for evaluating differences between two groups concerning continuous variables, while the χ^2^ test was employed for categorical variables. The assessment of associations between ADL and MDs was undertaken through a Pearson correlation analysis. Furthermore, a hierarchical multiple regression approach was employed to meticulously appraise the relationship between ADL and MDs. This analytical framework comprised three distinct sets of models, each constructed and fitted for specific purposes. In Model 1, demographic variables, including age and gender, were taken into account. Model 2 expanded the analytical scope by encompassing variables such as residence, smoking status, and drinking status. Finally, Model 3 included variables related to chronic health conditions, specifically hypertension, dyslipidemia, and diabetes. To ensure the robustness and reliability of the obtained results, a sensitivity analysis was meticulously conducted on the CHARLS dataset, meticulously considering its intricate sampling methodology. This focused analysis specifically examined the influence of age and the presence of chronic diseases, such as hypertension, dyslipidemia, and diabetes, on the established associations.

### 2.6 Machine learning algorithm

#### 2.6.1 Parameter setup

The research model was meticulously implemented utilizing the PyCharm 2023.1.2 integrated development environment, executed on a computer operating the Windows 11 operating system. The software development process was executed within the designated development environment, Anaconda, leveraging Python version 3.10.2 for coding and analysis. Notably, a seed value, referred to as random_state, was systematically initialized as the integer 42 to ensure reproducibility and consistency in the results. The experiment itself involved the division of the original dataset into two distinct subsets: a training set, which encompassed 70% of the initial dataset, and a testing set, constituting the remaining 30% of the original dataset. Subsequently, the assessment and evaluation of the model’s performance were conducted by scrutinizing the categorization outcomes that emanated from the testing dataset.

#### 2.6.2 Experimental models

##### 2.6.2.1 Support vector machine

Support Vector Machine (SVM) is a prominent supervised learning technique categorized as a generalized linear classifier. Its primary goal is to accurately categorize data by identifying the maximum-margin hyperplane as the decision boundary for the training dataset. SVM’s versatility is evident in its ability to perform nonlinear classification tasks through the use of kernel functions, making it a cornerstone of kernel-based learning. SVM is particularly adept at handling high-dimensional problems, including those with large feature spaces, and excels in managing complex relationships among nonlinear features (24, 25).

##### 2.6.2.2 Decision tree learning (DT)

Decision tree learning is a widely used supervised technique in statistics, data mining, and machine learning, valued for its applicability in classification and regression tasks. Decision trees are constructed as prediction models, extracting insights from observational data. In classification, decision trees function as tree models for discrete target variable values. Terminal nodes, or leaves, represent class labels, while branches delineate feature combinations leading to these labels. In regression decision trees, continuous values, typically real numbers, are accommodated for the target variable. These trees are versatile, handling various data types, including categorical sequences, enhancing their utility in diverse analytical contexts. The simplicity and interpretability of decision trees make them appealing within the machine learning community. Their visual and intuitive representation facilitates transparent decision-making processes. Decision trees also find utility in decision analysis, vividly portraying complex decision structures and associated decision-making processes. In the context of data mining, they serve as effective tools for describing data and extracting actionable insights to inform decision-making processes (26–28).

##### 2.6.2.3 Logistic regression

Logistic regression is a widely adopted methodology in supervised learning within machine learning (Equation 1, Logistic regression). Unlike linear regression, logistic regression primarily addresses classification problems, including multi-classification scenarios. In logistic regression, a training phase exposes the model to a dataset with n-groups, referred to as the training set. During this phase, the model assimilates patterns and relationships critical for making informed classification decisions. Post-training, the model’s acquired knowledge is applied to classify one or more datasets, known as the test set. These datasets consist of p indicators, each contributing to the model’s decision-making process. Logistic regression is a vital tool in supervised learning, effectively handling classification challenges. Its adaptability makes it a cornerstone of machine learning methodologies, with applications across diverse domains, including the scientific and medical fields (29, 30).

(1) $$sigmoid=\frac{1}{1+e^{-z}}$$

#### 2.6.3 Evaluating indicator

This study employed accuracy, precision, recall, and F1-score as the chosen evaluation metrics (31, 32).

Accuracy is a metric used to estimate the proportion of correctly labelled instances out of the total number of instances. The computation formula for accuracy is as follows (Equation 2):

(2) $$Accuracy=\frac{TP+TN}{TP+FP+TN+FN}$$

Precision refers to the proportion of positive cases in the prediction results that are accurately anticipated by the formula (Equation 3).

(3) $$Precision=\frac{TP}{TP+FP}$$

The recall metric measures the proportion of accurately predicted positive examples of genuine results, as determined by the following formula (Equation 4):

(4) $$Recall=\frac{TP}{TP+FN}$$

The F1-score is a metric that indicates the robustness of a model, as it considers both precision and recall rates. A higher value of F1 is indicative of superior model performance. The formula for calculation is as follows (Equation 5, F1-score):

(5) $$F1=\frac{2TP}{2TP+FN+FP}=\frac{2*Precision*Recall}{Precision+Recall}$$

TP = True Positive, FP = False Positive, FN = False Negative, TN = True Negative.

## 3 Results

### 3.1 Characteristics of samples

Exclusions were made among the enrolled participants, namely targeting individuals aged 45 and below, as well as those with inadequate demographic or health data. Consequently, the ultimate examination encompassed a total of 10,062 participants, consisting of 3,950 males (39.26%) and 6,112 females (60.74%). Table 2 provides a summary of the fundamental demographic characteristics of the sample population identified with MDs by medical professionals. The mean age of those diagnosed with MDs was 70.86 years, which was higher compared to the mean age of 64.35 years for those who were not diagnosed. Additionally, the mean BI for diagnosed individuals was 70.88, significantly lower than the mean score of 87.77 for undiagnosed individuals. Significant variations were noted in relation to gender, place of residence, smoking status, drinking status, and the prevalence of hypertension, diabetes, and dyslipidemia (*p* < 0.05). This finding suggests that individuals who are older and have lower levels of daily life functioning are more susceptible to developing MDs. The prevalence of MDs is influenced by various factors, including gender, place of residence, smoking status, alcohol use, hypertension, diabetes, and dyslipidemia.

**Table 2:** Demographic characteristics of middle-aged and older-adult Chinese with and without memory-related disease by doctor.

Variables	Not diagnosed with a memory-related illness by a doctor	Diagnosed with memory-related disease by a doctor	*p*-value	*t*/χ2
No. subjects (%)	9,695 (96.35)	367 (3.65)		
Age, year			<0.001	11.825
	64.35	70.86		
SD	9.91	10.37		
Sex, *n* (%)			0.002	9.924
Male	3,777 (38.96)	173 (47.14)		
Female	5,918 (61.04)	194 (52.86)		
Residence, *n* (%)			0.04	8.299
Central of city/town	1763 (18.18)	79 (21.53)		
Urban–rural integration zone	714 (7.37)	38 (10.35)		
Rural	7,187 (74.13)	249 (67.85)		
Special zone	31 (0.32)	1 (0.27)		
Education, *n* (%)				
Primary or below	7,252 (74.80)	277 (75.48)	0.694	0.731
Secondary school (Middle school + High school)	2,326 (23.99)	84 (22.89)		
College or above	117 (1.21)	6 (1.63)		
Smoking status, *n* (%)			<0.001	35.85
Yes	2,143 (22.10)	63 (17.17)		
Quit	1,380 (14.23)	93 (25.34)		
No	6,172 (63.66)	211 (57.49)		
Drinking status, *n* (%)			0.005	10.508
Drink more than once a month	1921 (19.81)	49 (13.35)		
Drink but less than once a month	638 (6.58)	21 (5.72)		
None	7,136 (73.61)	297 (80.93)		
Hypertension, *n* (%)				
Yes	1,262 (13.02)	230 (62.67)	<0.001	690.31
No	8,433 (86.98)	137 (37.32)		
Diabetes, *n* (%)				
Yes	744 (7.67)	66 (17.98)	<0.001	50.776
No	8,951 (92.33)	301 (82.02)		
Dyslipidemia, *n* (%)				
Yes	1,074 (11.08)	157 (42.78)	<0.001	330.969
No	8,621 (88.92)	210 (57.22)		
Activity of daily living (The Barthel Index)			<0.001	361.409
	87.77	70.88		
SD	15.90	28.18		

### 3.2 The correlation between covariates and MDs

To explore the intricate relationships between the presence of MDs and the independent as well as covariate variables, we employed Pearson correlation analysis as a rigorous analytical tool. The results of this analysis are comprehensively presented in Table 3, showcasing the correlation coefficients and their corresponding *p*-values.

**Table 3:** Correlation between covariates and memory-related disease.

Variables	Memory-related disease	
	*r*	*p*-value
Age	0.117	< 0.001
Gender	−0.31	0.002
Residence	−0.023	0.02
Smoking status	0.024	0.016
Drinking status	−0.032	0.001
Hypertension	0.262	< 0.001
Diabetes	0.071	< 0.001
Dyslipidemia	0.181	< 0.001
Activity of daily living (The Barthel Index)	−0.188	< 0.001

Memory-related disease: 0 = “Not diagnosed with a memory-related illness by a doctor,”1 = “Diagnosed with Memory-Related Disease by a Doctor”; Gender: 1 = “Male,”2 = “Female”; Residence: 1 = “Central of City/Town,”2 = “Urban–Rural Integration Zone,”3 = “Rural,”4 = “Special Zone”; Smoking status: 2 = “Yes,”1 = “Quit,”0 = “No”; Drinking status: 2 = “Drink more than once a month,”1 = “Drink but less than once a month,”0 = “No”; Hypertension/Diabetes/Dyslipidemia: 0 = “No,”1 = “Yes”.

Among the diverse factors scrutinized, our investigation revealed a noteworthy inverse association between the independent variable, ADL, and the diagnosis of MDs (*r* = −0.188, *p* < 0.001). This significant finding signifies that individuals with impaired abilities in performing their daily life activities are associated with an elevated susceptibility to MDs.

Furthermore, the examination of various covariate variables yielded compelling insights. Age exhibited a positive correlation with MDs (*r* = 0.117, *p* < 0.001), indicating that advanced age is linked to a positive correlation with an increased occurrence of MDs. Similarly, smoking status (*r* = 0.024, *p* = 0.016), hypertension (*r* = 0.262, *p* < 0.001), diabetes (*r* = 0.071, *p* < 0.001), and dyslipidemia (*r* = 0.181, *p* < 0.001) all demonstrated statistically significant positive associations with the occurrence of MDs. This suggests that individuals of older age, smokers, and those with underlying conditions such as hypertension, diabetes, and hyperlipidemia are positively correlated with a heightened occurrence of developing memory-related illnesses.

Conversely, substantial negative associations were identified between the presence of MDs and variables such as gender (*r* = −0.31, *p* = 0.002), place of residence (*r* = −0.023, *p* = 0.02), and drinking status (*r* = −0.032, *p* = 0.001). These findings elucidate that, within the parameters of our study, females exhibit a negative correlation with a reduced likelihood of receiving an MD diagnosis. Additionally, variations in the prevalence of MDs across different residential regions were observed, indicating the negative correlation and influence of geographical context on MD prevalence. Notably, individuals exhibiting lower alcohol consumption patterns appear to manifest a negative correlation with a higher propensity for MD diagnosis.

In sum, this comprehensive correlation analysis provides critical insights into the multifaceted interplay between MDs and various demographic and health-related factors, shedding light on positive and negative correlations associated with memory-related diseases and avenues for further exploration in the domain of memory-related disease research.

### 3.3 Associations between ADL and MDs

To better understand the association between MDs and ADL, we employed covariate control through three distinct models (Model 1, Model 2, Model 3). These models systematically explored associations between independent variables (ADL, Age, Gender, Residence, Smoking status, etc.) and the dependent variable, MDs.

In Model 1, adjusting for age and gender (*R* = 0.204, *F* = 145.908, *p* < 0.001), we found a significant negative correlation between ADL and MDs (*B* = −0.002, *β* = −0.172, *t* = −17.004, 95% CI = −0.002, −0.002, *p* = 0.000). This emphasizes the heightened vulnerability of individuals with compromised daily life abilities to memory-related illnesses. Model 2, with additional covariates (residence, smoking status, and alcohol consumption, *R* = 0.212, *F* = 47.372, *p* < 0.001), still showed a significant negative correlation between ADL and MDs (*B* = −0.002, *β* = −0.172, *t* = −16.800, 95% CI = −0.002, −0.002, *p* = 0.000). In the final iteration, Model 3, incorporating hypertension, dyslipidemia, and diabetes as covariates, continued to affirm the negative correlation between ADL and MDs (*B* = −0.002, *β* = −0.142, *t* = −14.393, 95% CI = −0.002, −0.001, *p* = 0.000), demonstrating an overall significant fit (*R* = 0.346, *F* = 104.887, *p* < 0.001).

All three models showed statistically significant associations between independent variables and the outcome (*p* < 0.001). The R value increased progressively from 0.204 in Model 1 to 0.346 in Model 3, indicating an enhanced capability to elucidate variability in the outcome. Despite covariate adjustments, all models consistently underscored the significant association between diminished daily living abilities and an elevated susceptibility to memory-related diseases, highlighting the robustness of this relationship, even when considering potential confounding factors (see Table 4).

**Table 4:** Associations between ADL and memory-related disease in middle-aged and older-adult Chines.

Model	*R*	*F*	*p*-value	Variables		*B*	*β*	*t*	95%CI		*p*-value
Model 1	0.204	145.908	<0.001		ADL	−0.002	−0.172	−17.004	−0.002	−0.002	0.000
					Age	0.001	0.071	7.008	0.001	0.002	0.000
				Gender	Male	0.012	0.032	3.298	0.005	0.020	0.001
					Female (Ref.)						
Model 2	0.212	47.372	<0.001		ADL	−0.002	−0.172	−16.800	−0.002	−0.002	0.000
					Age	0.001	0.067	6.624	0.001	0.002	0.000
				Gender	Male	0.018	0.046	3.162	0.007	0.029	0.002
					Female (Ref.)						
				Residence	Central of City/Town	0.004	0.007	0.108	−0.061	0.068	0.914
					Urban–Rural Integration Zone	0.010	0.014	0.299	−0.055	0.075	0.765
					Rural	−0.013	−0.032	−0.414	−0.077	0.050	0.679
					Special Zone (Ref.)						
				Smoking status	Yes	−0.007	−0.016	−1.229	−0.019	0.004	0.219
					Quit	0.008	0.014	1.152	−0.005	0.021	0.249
					No (Ref.)						
				Drinking status	Drink more than once a month	−0.013	−0.027	−2.426	−0.023	−0.002	0.015
					Drink less than once a month	−0.005	−0.006	−0.627	−0.019	0.010	0.531
					No (Ref.)						
Model 3	0.346	104.887	<0.001		ADL	−0.002	−0.142	−14.393	−0.002	−0.001	0.000
					Age	0.001	0.069	6.990	0.001	0.002	0.000
				Gender	Male	0.012	0.031	2.225	0.001	0.022	0.026
					Female (Ref.)						
				Residence	Central of City/Town	0.002	0.004	0.061	−0.060	0.063	0.951
					Urban–Rural Integration Zone	0.005	0.007	0.164	−0.057	0.068	0.870
					Rural	−0.010	−0.024	−0.324	−0.071	0.051	0.746
					Special Zone (Ref.)						
				Smoking status	Yes	−0.005	−0.010	−0.813	−0.016	0.007	0.416
					Quit	0.007	0.014	1.141	−0.005	0.020	0.254
					No (Ref.)						
				Drinking status	Drink more than once a month	−0.009	−0.019	−1.775	−0.019	0.001	0.076
					Drink less than once a month	−0.008	−0.010	−1.091	−0.022	0.006	0.275
					No (Ref.)						
				Hypertension	Yes	0.115	0.218	22.656	0.105	0.125	0.000
					No (Ref.)						
				Diabetes	Yes	0.014	0.020	2.067	0.001	0.026	0.039
					No (Ref.)						
				Dyslipidemia	Yes	0.072	0.126	12.963	0.061	0.083	0.000
					No (Ref.)						

To comprehensively address the potential influence of distinct age cohorts on the outcome variable, we thoughtfully stratified age into two discernible groups: the middle-aged cohort (≥45 years and < 60 years, as elaborated in Table 5) and the older-adult cohort (≥60 years, as expounded upon in Table 6). Through these meticulous age-stratified analyses, we endeavored to explore the intricate interplay between ADL and MDs across different generational segments.

**Table 5:** Associations between ADL and MDs in middle-aged cohort.

Model	*R*	*F*	*p*-value	Variables		*B*	*β*	*t*	95%CI		*p*-value
Model 1	0.171	33.899	<0.001		ADL	−0.001	−0.167	−9.830	−0.002	−0.001	0.000
					Age	0.000	0.008	0.471	−0.001	0.001	0.638
				Gender	Male	0.011	0.047	2.743	0.003	0.019	0.006
					Female (ref.)						
Model 2	0.185	11.994	<0.001		ADL	−0.001	−0.161	−9.358	−0.002	−0.001	0.000
					Age	0.000	0.006	0.332	−0.001	0.001	0.740
				Gender	Male	0.011	0.047	1.777	−0.001	0.023	0.076
					Female (Ref.)						
				Residence	Central of city/town	0.011	0.038	0.405	−0.042	0.063	0.686
					Urban–rural integration zone	0.023	0.058	0.850	−0.030	0.076	0.396
					Rural	0.009	0.038	0.354	−0.043	0.061	0.723
					Special zone (Ref.)						
				Smoking status	Yes	0.005	0.018	0.765	−0.008	0.018	0.444
					Quit	0.017	0.044	2.103	0.001	0.032	0.036
					No (Ref.)						
				Drinking status	Drink more than once a month	−0.016	−0.058	−2.928	−0.026	−0.005	0.003
					Drink less than once a month	0.001	0.002	0.101	−0.014	0.015	0.920
					No (Ref.)						
Model 3	0.247	16.884	<0.001		ADL	−0.001	−0.146	−8.539	−0.002	−0.001	0.000
					Age	0.000	0.007	0.427	−0.001	0.001	0.669
				Gender	Male	0.007	0.030	1.159	−0.005	0.019	0.246
					Female (ref.)						
				Residence	Central of city/town	0.007	0.026	0.280	−0.044	0.059	0.779
					Urban–rural integration zone	0.020	0.051	0.752	−0.032	0.073	0.452
					Rural	0.007	0.030	0.281	−0.044	0.059	0.778
					Special zone (ref.)						
				Smoking status	Yes	0.007	0.026	1.115	−0.005	0.020	0.265
					Quit	0.016	0.043	2.066	0.001	0.031	0.039
					No (ref.)						
				Drinking status	Drink more than once a month	−0.015	−0.054	−2.741	−0.025	−0.004	0.006
					Drink less than once a month	0.000	0.001	0.030	−0.014	0.014	0.976
					No (ref.)						
				Hypertension	Yes	0.041	0.124	7.290	0.030	0.052	0.000
					No (ref.)						
				Diabetes	Yes	0.000	−0.001	−0.035	−0.014	0.014	0.972
					No (ref.)						
				Dyslipidemia	Yes	0.029	0.088	5.155	0.018	0.041	0.000
					No (ref.)						

**Table 6:** Associations between ADL and MDs in older-adult cohort.

Model	*R*	*F*	*p*-value	Variables		*B*	*β*	*t*	95%CI		*p*-value
Model 1	0.187	80.208	<0.001		ADL	−0.002	−0.173	−13.936	−0.002	−0.002	0.000
					Age	0.001	0.039	3.127	0.000	0.002	0.002
				Gender	Male	0.013	0.030	2.451	0.003	0.023	0.014
					Female (ref.)						
Model 2	0.197	26.953	<0.001		ADL	−0.002	−0.174	−13.889	−0.002	−0.002	0.000
					Age	0.001	0.035	2.809	0.000	0.002	0.005
				Gender	Male	0.021	0.047	2.698	0.006	0.036	0.007
					Female (ref.)						
				Residence	Central of city/town	−0.013	−0.024	−0.239	−0.121	0.095	0.811
					Urban–rural integration zone	−0.009	−0.010	−0.156	−0.117	0.100	0.876
					Rural	−0.038	−0.076	−0.690	−0.145	0.069	0.490
					Special zone (Ref.)						
				Smoking status	Yes	−0.013	−0.026	−1.600	−0.030	0.003	0.110
					Quit	0.004	0.007	0.425	−0.014	0.021	0.671
					No (ref.)						
				Drinking status	Drink more than once a month	−0.011	−0.020	−1.474	−0.025	0.004	0.141
					Drink less than once a month	−0.009	−0.010	−0.787	−0.030	0.013	0.432
					No (ref.)						
Model 3	0.368	80.323	<0.001		ADL	−0.002	−0.139	−11.592	−0.002	−0.001	0.000
					Age	0.001	0.042	3.547	0.001	0.002	0.000
				Gender	Male	0.016	0.036	2.194	0.002	0.030	0.028
					Female (ref.)						
				Residence	Central of city/town	−0.004	−0.006	−0.069	−0.106	0.099	0.945
					Urban–rural integration zone	−0.007	−0.008	−0.125	−0.110	0.097	0.901
					Rural	−0.021	−0.043	−0.405	−0.123	0.081	0.685
					Special zone (ref.)						
				Smoking status	Yes	−0.011	−0.022	−1.427	−0.027	0.004	0.154
					Quit	0.004	0.006	0.425	−0.013	0.020	0.671
					No (ref.)						
				Drinking status	Drink more than once a month	−0.005	−0.009	−0.748	−0.019	0.008	0.455
					Drink less than once a month	−0.014	−0.016	−1.375	−0.035	0.006	0.169
					No (ref.)						
				Hypertension	Yes	0.146	0.247	21.095	0.132	0.159	0.000
					No (ref.)						
				Diabetes	Yes	0.019	0.024	2.098	0.001	0.037	0.036
					No (ref.)						
				Dyslipidemia	Yes	0.094	0.143	12.017	0.079	0.110	0.000
					No (ref.)						

Remarkably, within both the middle-aged group and the older-adult group, these focused analyses unveiled strikingly similar patterns. In the middle-aged cohort, a noteworthy negative correlation between ADL and MDs emerged as a consistent finding (*B* = −0.001, *β* = −0.167, *t* = −9.830, 95% CI = −0.002, −0.001, *p* = 0.000). Similarly, the older-adult cohort exhibited a robust negative association between ADL and MDs (*B* = −0.002, *β* = −0.173, *t* = −13.936, 95% CI = −0.002, −0.002, *p* = 0.000). These findings held true across an array of age-stratified models and maintained statistical significance (*p* < 0.001) without exception.

Crucially, the consistent demonstration of these negative associations in Model 1, Model 2, and Model 3 further underscores the resilience of this observed relationship, even when adjusting for an extensive array of covariates. These insightful results contribute substantively to our understanding of the multifaceted factors associated with MDs, enriching the body of knowledge surrounding this critical medical domain.

To thoroughly assess the impact of chronic diseases, including hypertension, diabetes, and dyslipidemia, on the outcome variable, we categorized them into two cohorts: the Chronic Disease Group (as detailed in Table 7), comprising individuals with one or more of these conditions, and the No Chronic Disease Group (explained in Table 8), consisting of individuals without these conditions. This categorization allowed a detailed analysis of the impact of chronic diseases on the interplay between ADL and MDs.

**Table 7:** Associations between ADL and MDs in the chronic disease group.

Model	*R*	*F*	*p*-value	Variables		*B*	*β*	*t*	95%CI		*p*-value
Model 1	0.287	82.733	<0.001		ADL	−0.003	−0.221	−11.785	−0.004	−0.003	0.000
					Age	0.004	0.135	7.166	0.003	0.005	0.000
				Gender	Male	0.022	0.036	1.982	0.000	0.044	0.048
					Female (Ref.)						
Model 2	0.299	27.202	<0.001		ADL	−0.003	−0.221	−11.613	−0.004	−0.003	0.000
					Age	0.004	0.128	6.800	0.003	0.005	0.000
				Gender	Male	0.037	0.060	2.228	0.004	0.069	0.026
					Female (ref.)						
				Residence	Central of city/town	−0.005	−0.006	−0.049	−0.193	0.184	0.961
					Urban–rural integration zone	0.001	0.001	0.011	−0.189	0.192	0.992
					Rural	−0.044	−0.068	−0.464	−0.232	0.143	0.643
					Special zone (ref.)						
				Smoking status	Yes	−0.025	−0.033	−1.370	−0.060	0.011	0.171
					Quit	0.014	0.017	0.728	−0.024	0.052	0.466
					No (ref.)						
				Drinking status	Drink more than once a month	−0.034	−0.043	−2.106	−0.066	−0.002	0.035
					Drink less than once a month	−0.014	−0.012	−0.651	−0.056	0.028	0.515
					No (ref.)						
Model 3	0.389	37.815	<0.001		ADL	−0.003	−0.194	−10.530	−0.004	−0.002	0.000
					Age	0.004	0.124	6.777	0.003	0.005	0.000
				Gender	Male	0.016	0.027	1.013	−0.015	0.048	0.311
					Female (ref.)						
				Residence	Central of city/town	−0.014	−0.019	−0.150	−0.196	0.168	0.881
					Urban–rural integration zone	−0.017	−0.016	−0.185	−0.202	0.167	0.853
					Rural	−0.049	−0.075	−0.533	−0.230	0.132	0.594
					Special zone (ref.)						
				Smoking status	Yes	−0.013	−0.018	−0.752	−0.047	0.021	0.452
					Quit	0.023	0.028	1.237	−0.013	0.060	0.216
					No (ref.)						
				Drinking status	Drink more than once a month	−0.034	−0.042	−2.159	−0.064	−0.003	0.031
					Drink less than once a month	−0.019	−0.016	−0.914	−0.060	0.022	0.361
					No (ref.)						
				Hypertension	Yes	0.170	0.284	13.218	0.145	0.196	0.000
					No (ref.)						
				Diabetes	Yes	0.062	0.094	4.653	0.036	0.088	0.000
					No (ref.)						
				Dyslipidemia	Yes	0.125	0.207	10.131	0.101	0.149	0.000
					No (ref.)						

**Table 8:** Associations between ADL and MDs in the no chronic disease group.

Model	*R*	*F*	*p*-value	Variables		*B*	*β*	*t*	95%CI		*p*-value
Model 1	0.126	39.124	<0.001		ADL	−0.001	−0.101	−8.385	−0.001	−0.001	0.000
					Age	0.000	0.046	3.800	0.000	0.001	0.000
				Gender	Male	0.008	0.033	2.829	0.002	0.013	0.005
					Female (ref.)						
Model 2	0.128	12.110	<0.001		ADL	−0.001	−0.101	−8.238	−0.001	−0.001	0.000
					Age	0.000	0.045	3.712	0.000	0.001	0.000
				Gender	Male	0.008	0.037	2.138	0.001	0.016	0.033
					Female (ref.)						
				Residence	Central of city/town	0.007	0.025	0.310	−0.038	0.053	0.757
					Urban–rural integration zone	0.013	0.030	0.558	−0.033	0.060	0.577
					Rural	0.006	0.023	0.254	−0.040	0.051	0.799
					Special zone (ref.)						
				Smoking status	Yes	−0.003	−0.010	−0.610	−0.011	0.006	0.542
					Quit	0.001	0.004	0.269	−0.008	0.011	0.788
					No (ref.)						
				Drinking status	Drink more than once a month	0.000	0.000	−0.003	−0.007	0.007	0.998
					Drink less than once a month	−0.005	−0.011	−0.888	−0.015	0.006	0.374
					No (ref.)						

In the Chronic Disease Group, a statistically significant inverse relationship between ADL and MDs was evident (*B* = −0.004, *β* = −0.194, *t* = −10.530, 95% CI = −0.004, −0.002, *p* = 0.000). Surprisingly, this inverse association persisted in the No Chronic Disease Group, comprising individuals without these chronic conditions (*B* = −0.001, *β* = −0.101, *t* = −8.238, 95% CI = −0.001, −0.001, *p* = 0.000). These findings highlight that both ADL and age influence susceptibility to MDs, regardless of chronic afflictions (*p* < 0.001). Additionally, gender played a significant role in influencing this association in specific situations.

### 3.4 Machine learning algorithm confirm the link between ADL and MDs

Building upon the foundations laid by our statistical inquiry, which delineated a significant association between ADL and the occurrence of MDs, we embarked on a validation journey employing three robust machine learning models: SVM, DT, and LR. Our primary hypothesis posited that individuals with diminished ADL capabilities would indeed be associated with an elevated likelihood of experiencing memory-related afflictions. To put this hypothesis to the test, we meticulously scrutinized the predictive prowess of these machine learning paradigms.

**Figure 1:**
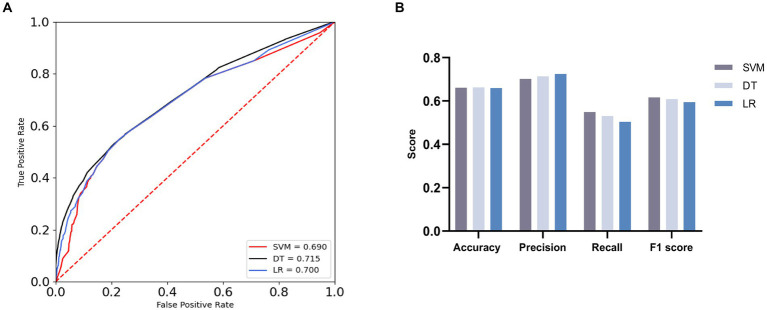
ROC curves and evaluation indicators [**(A)** ROC curves with AUC for the three machine learning algorithms. **(B)** Comparison of three machine learning algorithms].

The validation results (Figure 1A) revealed that both DT and LR achieved Area Under the Receiver Operating Characteristic Curve (AUC) values above the critical threshold of 0.7, specifically reaching 0.715 and 0.700, respectively. This underscores the substantial diagnostic utility embedded in the DT and LR models. Furthermore, the SVM model displayed a commendable AUC of 0.69, surpassing the critical threshold of 0.5, affirming its discernible diagnostic capability.

A comprehensive assessment of the three machine learning models, including evaluation metrics such as Accuracy, Precision, Recall, and F1 Score, followed (Figure 1B; Table 9). The models demonstrated comparable performance in Accuracy, with SVM achieving an estimated accuracy of 0.661, DT slightly outperforming at approximately 0.662, and LR displaying a marginally lower accuracy of around 0.659. This convergence suggests analogous levels of predictive accuracy.

**Table 9:** Comparison of three machine learning algorithms.

Model	Accuracy	Precision	Recall	F1 score
SVM	0.661	0.702	0.549	0.616
DT	0.662	0.714	0.531	0.609
Lr	0.659	0.725	0.503	0.594

In Precision, LR emerged as the front-runner, boasting a precision rate of approximately 0.725, followed closely by DT with a precision rate of roughly 0.714, and SVM, which garnered a precision rate of approximately 0.702. This underscores LR’s superior ability in accurately classifying positive cases.

In Recall, SVM exhibited the highest recall rate at approximately 0.531, followed by DT at approximately 0.549, and LR, achieving a recall rate of around 0.504. DT’s superior performance in identifying positive samples is conspicuous.

The F1 Score, adept at harmonizing Precision and Recall, particularly in datasets with skewed class distributions, provided valuable insights. LR, despite exhibiting the lowest score of roughly 0.5941, was succeeded by DT, with a slightly higher score of approximately 0.609. Notably, SVM demonstrated significantly superior performance, securing an approximate score of 0.616.

In conclusion, the comprehensive statistical findings, harmoniously corroborated by the machine learning model validations and AUC assessments, resoundingly affirm the existence of a substantial association between ADL and the occurrence of MDs. This correlation resurfaces consistently across both our initial statistical exploration and the subsequent validation via machine learning methodologies. These robust findings underscore the heightened association of MDs with individuals grappling with diminished daily living abilities, fostering a deeper understanding of this intricate interplay.

To enhance the credibility of the association between ADL and MDs, we strategically divided our dataset into two groups with distinct characteristics. The first group included the BI along with additional covariates, while the second group excluded the BI but retained the same set of covariates. This division aimed to mitigate potential confounding influences and strengthen the investigated relationship.

**Figure 2:**
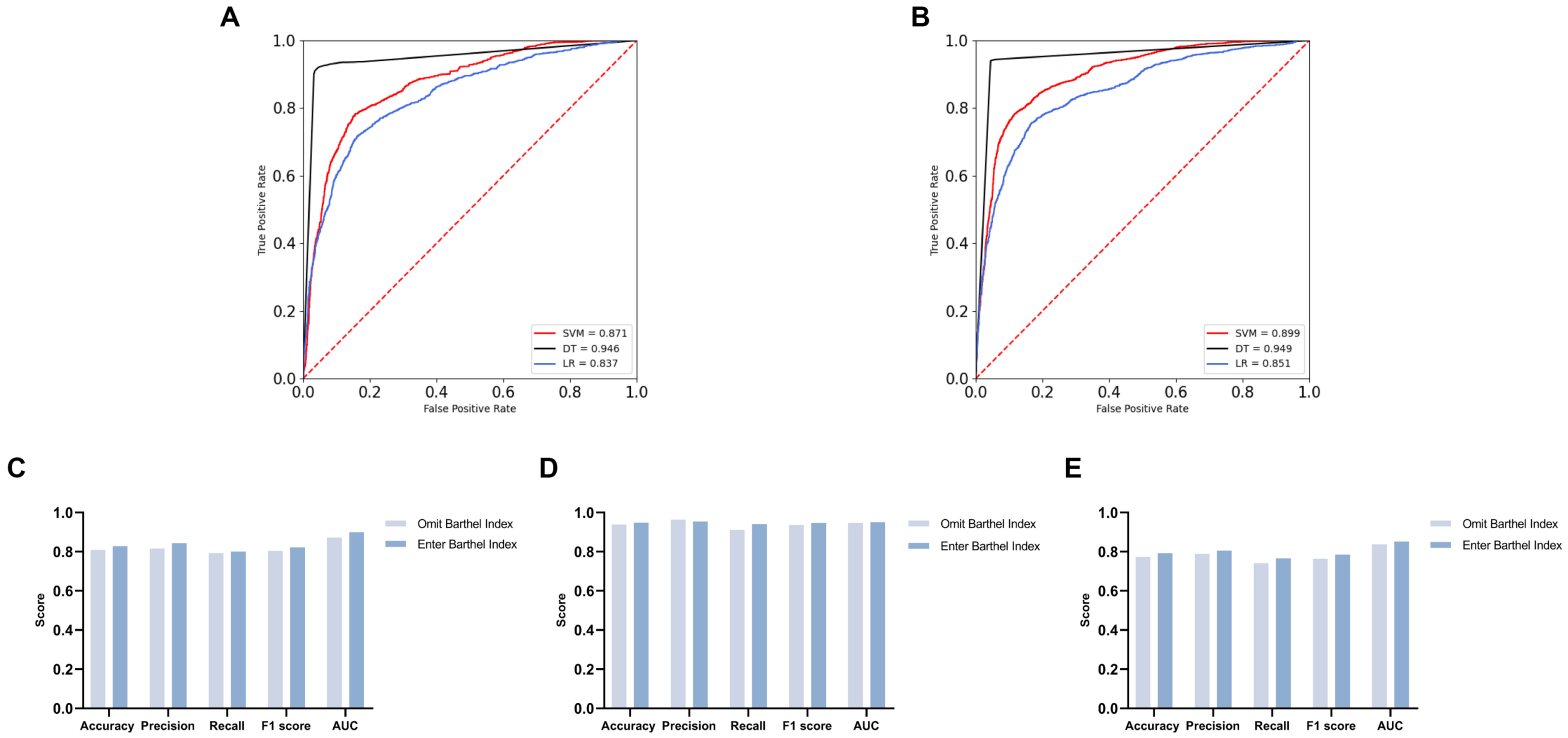
Comparison of overall effects of machine learning algorithms for two distinct datasets [**(A)** ROC curves with AUC for omit Barthel Index group. **(B)** ROC curves with AUC for enter Barthel Index group. **(C)** Contrasting the SVM model across the two groups. **(D)** Contrasting the DT model across the two groups. **(E)** Contrasting the LR model across the two groups].

Machine learning algorithms underwent rigorous validation for these two data groups. Findings revealed disparities in the efficacy of the three models across these subsets, as detailed in Figure 2 and Table 10. Importantly, with the inclusion of the BI, the SVM, DT, and LR models showed an augmentation in their AUC values. Specifically, AUC values for the SVM, DT, and LR models increased from 0.871, 0.946, and 0.837 to elevated values of 0.899, 0.949, and 0.851, respectively. This enhancement in AUC values indicates an overall improvement in the models’ performance, visually depicted in Figures 2A,B.

**Table 10:** Comparison of overall effects of machine learning algorithms for two distinct datasets.

Model	Accuracy		Precision		Recall		F1 score		AUC	
	Omit BI	Enter BI	Omit BI	Enter BI	Omit BI	Enter BI	Omit BI	Enter BI	Omit BI	Enter BI
SVM	0.808	0.828	0.816	0.844	0.792	0.8	0.804	0.822	0.871	0.899
DT	0.938	0.948	0.963	0.954	0.91	0.94	0.936	0.947	0.946	0.949
Lr	0.773	0.792	0.788	0.805	0.74	0.766	0.763	0.785	0.837	0.851

Further analysis suggests that the inclusion of the BI elevated the models’ predictive capabilities, as shown in Figures 2C–E. Among these models, the DT model emerged as the top performer, achieving high values of 0.948 in accuracy, 0.954 in precision, 0.940 in recall, and 0.947 in the F1 score, showcasing robust overall performance.

Together, AUC scores and the assessment metrics provide compelling evidence supporting the proposition that the inclusion of the BI enhances model performance. This reinforces the relationship between ADL and the diagnosis of memory-related disorders, affirming our initial hypotheses with unwavering conviction.

## 4 Discussion

In the backdrop of an unprecedented global demographic shift characterized by a burgeoning older-adult population, the burden imposed by MDs on society and households is mounting at an alarming rate (33). Therefore, the imperative of early intervention into factors intricately linked with MDs assumes paramount significance.

In the course of this comprehensive investigation, we embarked upon a nuanced exploration, unveiling an initially conspicuous reduction in the mean BI scores among the MDs-afflicted cohort, in stark contrast to their non-afflicted counterparts within the demographic landscape. Pearson correlation analysis corroborated the existence of an inverse relationship between the two. Following meticulous adjustments for covariates spanning age, gender, lifestyle preferences, and chronic comorbidities, ADL persisted in manifesting a pronounced negative correlation with MDs. Consequently, we proffer the supposition that compromised ADL is predisposing individuals to an elevated susceptibility to MDs. Machine learning algorithms, thoughtfully harnessed for validation, further reinforced our conjectures. In an independent experiment employing BI data exclusively, the discernible diagnostic value reflected in the Area AUC score substantiated our hypotheses. In comparative evaluations, pitting covariates in isolation against covariates concomitantly partnered with BI, the incorporation of BI precipitated a demonstrable augmentation in the overall predictive prowess of the model. This substantiated our underlying premise that attenuated ADL was associated with an escalated likelihood of MDs.

Notably, the influence of other covariates on MDs was manifest in our findings. Age, hypertension, diabetes, dyslipidemia, and smoking status surfaced with positive correlations with MDs, harmonizing with antecedent research tenets (34–37). However, our data unveiled an intriguing incongruity concerning alcohol consumption, contravening prior studies by revealing that individuals with lower alcohol intake were associated with a heightened likelihood of MDs (38). This disparity may potentially be ascribed to an overrepresentation of female participants in our cohort. Females, as a general trend, constitute a lesser proportion of habitual alcohol consumers (39, 40), thereby potentially introducing a gender-related bias into the results. Furthermore, the intricate influence of place of residence on MDs emerged as a multifaceted latent determinant (41), evading facile characterization through mere correlation. Future research endeavors may benefit from amalgamating additional socioeconomic variables, including retirement income, filial support, national safeguards, and community-based services, to unravel the multidimensional tapestry of MDs etiology.

In the purview of the present study, the negative correlation between ADL and MDs remained resolute, even following meticulous covariate adjustments, an association incontrovertibly validated by machine learning algorithms. Hence, the imperative of fortifying ADL among the middle-aged and older-adult, particularly those grappling with underlying chronic conditions, assumes pivotal significance (42, 43). Enhancing ADL not only underpins an augmented quality of life but also embodies the realization of the time-honored concept of “prevention before illness” within the realm of clinical medicine (44). For those already ensnared by the clutches of MDs, bolstering their self-care proficiency constitutes a pivotal strategy for retarding the inexorable march of disease progression (45, 46). These individual advancements bear the potential to alleviate the colossal burdens borne by families and society at large.

Nonetheless, it is incumbent upon us to acknowledge the inherent limitations permeating this study. Firstly, as a cross-sectional analysis, our endeavors were inherently incapable of tracking longitudinal changes within the sample, thereby precluding the validation of a causal relationship between ADL and MDs. It remains plausible that diminished ADL may emerge as a consequence of MDs *per se*. Thus, our current conclusions can merely assert a substantiated negative correlation between ADL and MDs. Secondly, within the vast expanse of machine learning algorithms, our study availed itself of a modest triad, without embarking upon an exhaustive comparative exploration to discern the most fitting model. Notwithstanding this, our comparative analysis retains meaningful relevance (47, 48). Lastly, the assessment of ADL hinged upon the BI, meticulously computed based on corresponding items nestled within the CHARLS dataset and subsequently derived from self-reports. While nuances do exist between self-reported and clinically ascertained data, certain studies resonate with the reliability of self-reported ADL assessments (49–52). Thus, the findings borne of this research, framed within the realm of scientific rigor, retain substantial significance.

## 5 Conclusion

In summary, this study constitutes a valuable addition to the growing body of evidence reaffirming the inverse relationship between ADL and MDs. The elucidated findings emphatically underscore that diminished ADL levels portend an augmented vulnerability to the onset of MDs.

## Data availability statement

The original contributions presented in the study are included in the article/supplementary material, further inquiries can be directed to the corresponding author.

## Ethics statement

The studies involving humans were approved by Ethical Review Committee at Peking University at: https://charls.charlsdata.com/pages/Data/2018-charls-wave4/zh-cn.html. The studies were conducted in accordance with the local legislation and institutional requirements. Written informed consent for participation was not required from the participants or the participants’ legal guardians/next of kin in accordance with the national legislation and institutional requirements.

## Author contributions

JH: Data curation, Methodology, Software, Writing – original draft. WW: Software, Writing – original draft. SW: Supervision, Writing – original draft. MG: Supervision, Writing – original draft. ZS: Supervision, Writing – review & editing. SC: Funding acquisition, Resources, Supervision, Writing – review & editing.

## Footnotes

^1^ http://charls.pku.edu.cn
